# Post-marathon Decline in Right Ventricular Radial Motion Component Among Amateur Sportsmen

**DOI:** 10.3389/fphys.2021.811764

**Published:** 2022-01-10

**Authors:** Zuzanna Lewicka-Potocka, Anna Maria Kaleta-Duss, Ewa Lewicka, Marcin Kubik, Anna Faran, Paweł Szymeczko, Rafał Gała̧ska, Grzegorz Raczak, Alicja Da̧browska-Kugacka

**Affiliations:** ^1^Department of Cardiology and Electrotherapy, Faculty of Medicine, Medical University of Gdańsk, Gdańsk, Poland; ^2^First Department of Cardiology, Faculty of Medicine, Medical University of Gdańsk, Gdańsk, Poland; ^3^Institute for Radiology, Cantonal Hospital Aarau, Aarau, Switzerland

**Keywords:** marathon run, amateur runners, revision method, right ventricular motion components, right ventricular dysfunction (RV dysfunction), 3D echocardiography, galectine-3, overtraining

## Abstract

Moderate physical activity has a positive impact on health, although extreme forms of sport such as marathon running may trigger exercise-induced cardiac fatigue. The explicit distinction between the right ventricular (RV) physiological response to training and maladaptive remodeling has not yet been determined. In this study, we aimed to analyze the impact of running a marathon on RV mechanics in amateur athletes using three-dimensional (3D) echocardiography (ECHO) and the ReVISION method (RV separate wall motion quantification). A group of 34 men with a mean age of 40 ± 8 years who successfully finished a marathon underwent ECHO three times, i.e., 2 weeks before the marathon (stage I), at the marathon finish line (stage II), and 2 weeks after the marathon (stage III). The ECHO findings were then correlated with the concentrations of biomarkers related to myocardial injury and overload and also obtained at the three stages. On finishing the marathon, the amateur athletes were found to have a significant (*p* < 0.05) increase in end-diastolic (with a median of 51.4 vs. 57.0 ml/m^2^) and end-systolic (with a median of 24.9 vs. 31.5 ml/m^2^) RV volumes indexed to body surface area, reduced RV ejection fraction (RVEF) (with a median of 51.0% vs. 46.0%), and a decrease in RV radial shortening [i.e., radial EF (REF)] (with a mean of 23.0 ± 4.5% vs. 19.3 ± 4.2%), with other RV motion components remaining unchanged. The post-competition decrease in REF was more evident in runners with larger total volume of trainings (*R*^2^ = 0.4776, *p* = 0.0002) and higher concentrations of high-sensitivity cardiac troponin I (*r* = 0.43, *p* < 0.05) during the preparation period. The decrease in REF was more prominent in the training of marathoners more than 47 km/week. At stage II, marathoners with a more marked decrease in RVEF and REF had higher galectin-3 (Gal-3) levels (*r* = −0.48 and *r* = −0.39, respectively; *p* < 0.05). Running a marathon significantly altered the RV performance of amateur athletes. Transient impairment in RV systolic function resulted from decreased radial shortening, which appeared in those who trained more extensively. Observed ECHO changes correlated with the concentrations of the profibrotic marker Gal-3.

## Introduction

Regular physical activity has many well-documented health benefits (Nystoriak and Bhatnagar, 2018), particularly a reduction in all-cause mortality (Pelliccia et al., 2021). In recent years, the growing popularity of long-distance running competitions has been observed (Nikolaidis et al., 2018). Although the minimum weekly amount of moderate- and vigorous-intensity exercise for healthy adults is known (Pelliccia et al., 2021), the beneficial upper level of sports activity is not defined. Undoubtedly, preparing to run a marathon requires many hours of intense training. Not only a challenging training regimen but also participating in the competition itself is exhausting and can result in cardiac fatigue (Oxborough et al., 2010).

During extreme training, due to the different compensatory capacities of the pulmonary vascular bed and systemic circuit, the increase in pulmonary artery pressure is disproportionately greater, and exercise-induced overload predominantly affects the right ventricle (RV) (La Gerche et al., 2014). Among acute manifestations, impaired relaxation and decreased contractility of the RV have been observed in marathon runners after competing (Elliott and La Gerche, 2015; Lewicka-Potocka et al., 2020). Some researchers even suggested that there is a risk of developing “exercise-induced arrhythmogenic RV cardiomyopathy,” among possible long-term consequences of repetitive bouts of intensive training (Heidbuchel et al., 2012). Nevertheless, there is still ongoing discussion about the boundary between physiological adaptation to exercise and pathological RV remodeling (Leischik et al., 2020).

Due to the complex anatomy and mechanics of contraction, the RV remains a diagnostic challenge. Following the anatomical axes, overall RV systolic movement can be divided into three components, namely, longitudinal (displacement of the tricuspid annulus toward the apex), anteroposterior (stretching the RV wall by the contraction of the septum), and radial (internal relocation of the RV free wall) (Lakatos et al., 2017). Each of these components and their contraction can be precisely assessed with the advanced ECHO technique, i.e., the ReVISION method (RV separate wall motion quantification) (Lakatos et al., 2017). This approach overcomes the limitations of conventional parameters and allows precise estimation of all RV motion components and their contribution to global RV function.

Normally, the relative participation of these motion components in global RV function is rather equal, although it may vary depending on age and underlying diseases (Lakatos et al., 2017, 2020). Although current literature on this topic is scarce, it seems that changes in RV motion contribution or reduction in even single motion displacement can be an early marker of myocardial damage.

On the biochemical level, the increased concentrations of biomarkers reflecting myocardial overload and injury can be found after a marathon run (La Gerche et al., 2012). We observed a post-run increase in the plasma levels of high-sensitivity cardiac troponin I (hs-cTnI), heart-type fatty acid binding protein (H-FABP), N-terminal proatrial natriuretic peptide (NT-proANP), B-type natriuretic peptide (BNP), growth differentiation factor 15 (GDF-15), and galectin-3 (Gal-3) (Kaleta-Duss et al., 2020).

In this study, we aimed to analyze the impact of running a marathon on the mechanics of RV contraction in amateur athletes using three-dimensional (3D) echocardiography (ECHO) and the ReVISION method. We hypothesized that intense endurance exercise results in cardiac dysfunction that predominantly affects the RV and may be affected by the prerace training intensity and reflected by the increment of cardiac biomarkers of myocardial injury and overload.

## Materials and Methods

### Participants

Runners, who planned to attend the 2nd PZU Marathon in Gdańsk, Poland, were recruited to the study *via* invitations sent to local sports clubs. Due to the fact that there are known significant gender-linked differences in the structure of the heart of both male and female athletes (Di Paolo and Pelliccia, 2007) to avoid eventual non-homogeneity of the studied group, we decided to include only men. In the pre-participation screening, training habits and medical history were collected from every volunteer. Eligible candidates had to be aged between 20 and 55 years with no concomitant chronic diseases. We included runners who trained ≤11 h/week.

### Study Design

The study was conducted in three stages. Physical, ECG, and echocardiographic examinations were performed at each stage, and venous blood samples were collected to test the concentration of the selected laboratory parameters and cardiovascular biomarkers. The analysis took place 2 weeks before the marathon (stage I), at the marathon finish line (stage II), and 2 weeks after the marathon (stage III). Additionally, at stage I, a cardiopulmonary exercise test (CPET) was performed, and the methodology has been presented in detail previously (Lewicka-Potocka et al., 2021). The study protocol was approved by the Independent Bioethics Commission for Research of the Medical University of Gdansk (NKBBN/104/2016), and all participants gave written informed consent.

### Measurements

The ECHO was performed with a Vivid E9 apparatus (GE Healthcare, Horten, Norway) equipped with a 4VD transducer. In line with the current recommendations (Lang et al., 2015), standard 2D views and measurements were obtained and analyzed off-line with echocardiographic quantification software (EchoPac 201, GE Healthcare). The methodology for the echocardiographic measurements, such as 2D parameters reflecting the RV systolic function, has been described in detail previously (Lewicka-Potocka et al., 2020, 2021). In those manuscripts, we analyzed the marathon impact on conventional RV measures such as tricuspid annular plane systolic excursion (TAPSE), fractional area change (FAC), tricuspid lateral annular systolic velocity (S′ wave), four-chamber longitudinal strain, and diastolic RV function including the RV isovolumetric relaxation time.

All participants underwent 3D ECHO in accordance with the guidelines (Lang et al., 2012), and ECG-triggered multiple-beat full-volume 3D data sets for the left ventricle (LV) and RV were acquired from the apical view while participants held their breath for 5 s. Further analysis was performed off-line using dedicated commercially available systems on an EchoPac workstation with integrated software for LV and RV analysis (4D RV-Function 2, TomTec, Imaging GmbH, Unterschleissheim, Germany). The end-diastolic and end-systolic volumes (EDV and ESV) of the LV and RV indexed to body surface area were calculated together with the stroke volume (SV) and ejection fraction (EF). Additionally, a detailed analysis of RV mechanics was performed using the ReVISION method (Lakatos et al., 2017). Along known RV motion directions, three volumetric components were specified in the semiautomatically exported 3D RV beutel, and their volume changes over time were calculated following orthogonal anatomical axes. The volumes contributing to only one motion direction were measured at each time, while the others were blocked. Thus, the global RV function was decomposed and separate EFs such as longitudinal (LEF), radial (REF), and anteroposterior (APEF) were obtained. The relative contributions of the motion components to global RVEF were also determined and presented as indexes such as LEFi (LEF/RVEF), REFi (REF/RVEF), and APEFi (APEF/RVEF).

### Biochemical Analysis

The concentrations of selected cardiovascular biomarkers, which were proved to be elevated after marathon run (Kaleta-Duss et al., 2020), such as hs-cTnI, H-FABP, NT-proANP, BNP, GDF-15, and Gal-3 were used to perform correlations with echocardiographic parameters. The methodology description of obtaining these biomarkers has been presented in detail previously (Kaleta-Duss et al., 2020).

### Statistical Analysis

All statistical analyses were performed using Statistica 13.3 software (StatSoft Inc., Tulsa, OK, USA) and R version 4.0.4 (https://cran.r-project.org/). The normal distribution of data was checked using the Shapiro–Wilk test. The normally distributed data are presented as mean ± SD, while those diverging from normal distribution by means of the median with respective interquartile range (IQR).

A comparison between the three stages of the study, such as LV and RV volumes and EFs, was performed using ANOVA and Tukey's *post hoc* test for normally distributed data or a Friedman test and *post hoc* test for non-normally distributed variables.

A comparison of RV motion components and their ratios among the study stages was performed using repeated-measures ANOVA with subsequent Tukey's *post hoc* test for multiple comparisons. The assumptions of the analysis were checked using Mauchly's test of sphericity. The linear regression analysis was performed to determine the relationship of ECHO findings with the cardiovascular biomarkers obtained at each stage of the study (I–III), such as hs-cTnI, H-FABP, NT-proANP, BNP, GDF-15, and Gal-3. The relationships between the difference of REF at stages I and II and the training volume (expressed as either the number of kilometers or hours per week) were modeled using the linear regression or non-linear second-order function estimation, and the goodness of fit was inferred based on the respective coefficient of determination (*R*^2^). To provide results reflecting a larger population, the relationship analysis of the ECHO findings with the training habits was performed excluding the definitely outstanding values. A *p*-value < 0.05 was considered significant.

## Results

In total, we enrolled 34 eligible amateur runners who successfully finished the marathon competition. The participants were Caucasian men with a mean age of 40 ± 8 years. The characteristics of the study group have been previously presented thoroughly, such as their training habits and results of CPET (Lewicka-Potocka et al., 2021). The mean achieved marathon finishing time was 3.7 ± 0.4 h. When preparing for a marathon, the mean reported training distance was 56.5 ± 19.7 km/week, and the mean total training time was 6.48 ± 2.3 h/week, whereas the peak oxygen uptake (VO_2_peak) was 53.7 ± 6.9 ml/kg/min (Lewicka-Potocka et al., 2021). The results on the biochemical analysis between study stages and changes in the concentrations of cardiovascular biomarkers (hs-cTnI, Gal-3, H-FABP, NT-proANP, BNP, and GDF-15) expressed as mean ± SD have been described in detail recently (Kaleta-Duss et al., 2020). Concerning correlations between biomarkers and ECHO parameters presented in this study, the mean concentration of Gal-3 at stage I was 8.53 ± 3.04 ng/ml vs. 10.65 ± 2.33 ng/ml at stage II. The mean level of H-FABP obtained after the marathon was 13.57 ± 9.63 ng/ml. At stage I, the mean concentration of hs-cTnI was 0.01 ± 0.01 ng/ml with 0.06 ± 0.09 ng/ml at the marathon finish line (Kaleta-Duss et al., 2020).

**Figure 1:**
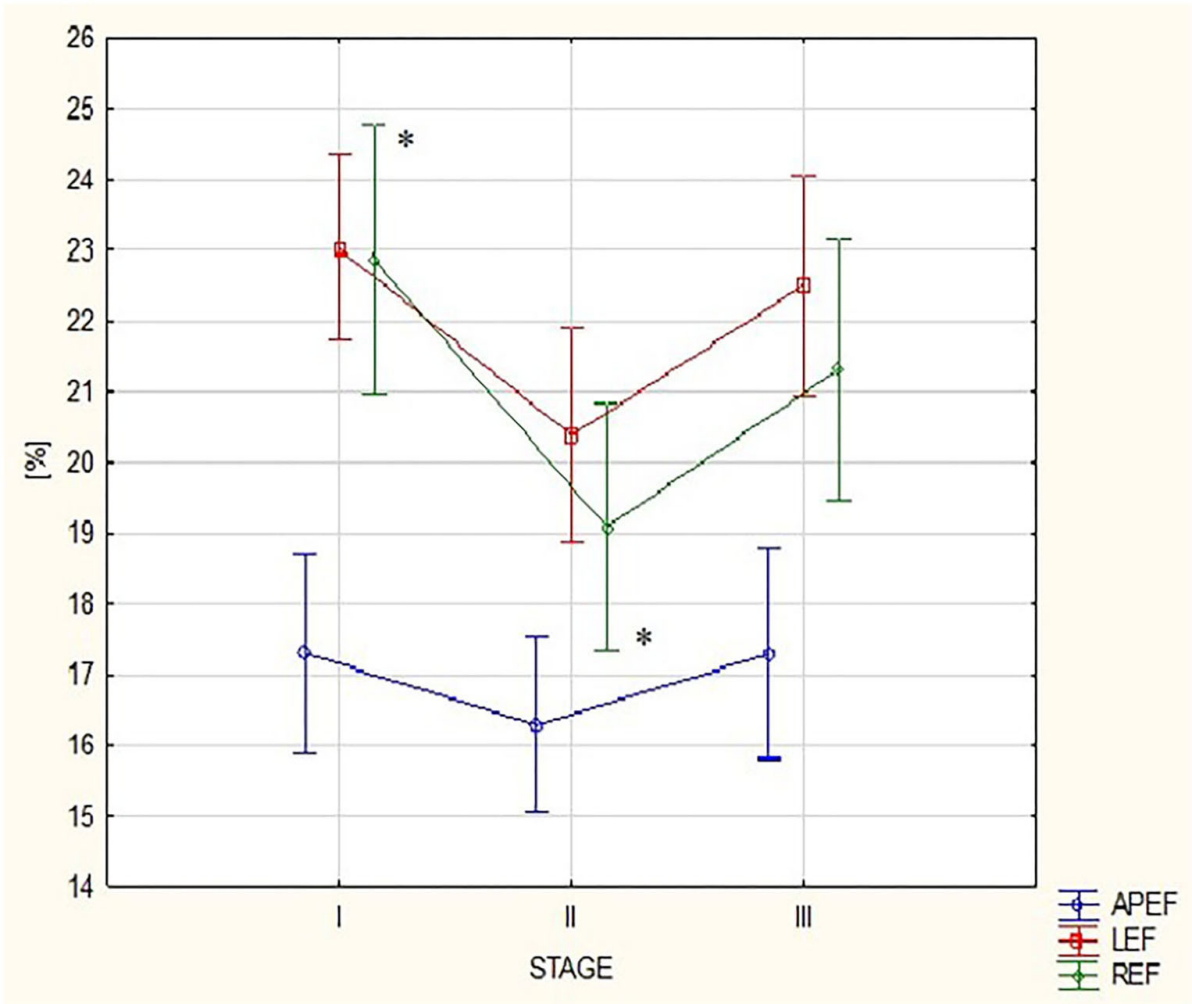
Changes in right ventricular (RV) motion components: anteroposterior ejection fraction (EF), longitudinal EF, and radial EF between the three study stages. APEF, anteroposterior ejection fraction; LEF, longitudinal ejection fraction; REF, radial ejection fraction; Stage I-2 weeks before marathon; Stage II-at marathon finish line; Stage III-2 weeks after marathon. *For REF: ANOVA *p*-value < 0.05, *post hoc* Tuckey test *p*-value < 0.05.

Table 1 presents 3D ECHO RV and LV parameters obtained in the three stages of the study, such as volumes and EFs. A detailed analysis of the RV motion components is shown in Table 2. The comparison of results from stages I and III revealed no differences (Tables 1, 2). After the run, a significant increase in the RV volumes, such as RVEDV and RVESV, was observed with a reduction in LVEDV (Table 1). The marathon competition resulted in significant deterioration of RV systolic function, with no influence on LVEF (Table 1). The post-run decrease in RV radial contraction, expressed by REF, was the main contributor to the exercise-induced drop in global RVEF (Table 2; Figure 1). No significant post-run changes in longitudinal or anteroposterior EFs were noticed (Figure 1). The reduction in radial shortening was transient and normalized within a 2-week interval. The contraction pattern and relative contribution of all RV motion components to global RV systolic function, quantified as LEFi, REFi, and APEFi, did not differ between study stages (Table 2).

**Table 1:** Three-dimensional (3D) echocardiographic parameters of right and left ventricle obtained in amateur marathon runners.

**Parameter**	**Stage I**	**Stage II**	**Stage III**	**ANOVA**	***Post-hoc P*** **-value**	
	**Mean** **±** **SD^a^** **or Median (1st; 3rd quartile)^b^**			***P*-value**	**Stage I vs. II**	**Stage I vs. III**
RVEDV [ml/m^2^]	51.4 (44.9;58.5)	57.0 (52.0;61.6)	52.6 (47.5;58.4)	**∧ <0.05**	**<0.05**	>0.05
RVESV [ml/m^2^]	24.9 (21.3;29.1)	31.5 (27.5;34.4)	26.8 (23.0;29.2)	**∧ <0.05**	**<0.05**	>0.05
RVSV [ml]	53.0 (46.0;59.0)	53.0 (48.8;57.0)	53.0 (46.0;59.0)	∧>0.05	–	–
RVEF [%]	51.0 (50.0;53.0)	46.0 (43.0;48.3)	51.0 (48.0;53.0)	**∧ <0.05**	**<0.05**	>0.05
LVEDV [ml/m^2^]	57.2 ± 10.4	52.0 ± 8.7	57.2 ± 11.0	^*^ <0.05	**<0.05**	>0.05
LVESV [ml/m^2^]	25.1 (20.6;28.0)	22.9 (21.2;26.5)	24.4 (20.9;27.8)	∧>0.05	–	–
LVSV [ml]	63.7 ± 11.7	55.0 ± 7.8	63.8 ± 13.4	^*^ <0.05	**<0.05**	>0.05
LVEF [%]	56.4 ± 3.6	54.9 ± 4.6	56.4 ± 3.4	^*^>0.05	–	–

*Stage I, 2 weeks before the marathon run; Stage II, at the finish line; Stage III, 2 weeks after the competition; SD, standard deviation; RVEDV, right ventricular end-diastolic volume indexed to body surface area; RVESV, right ventricular end-systolic volume indexed to body surface area; RVSV, right ventricular stroke volume; RVEF, right ventricular ejection fraction; LVEDV, left ventricular end-diastolic volume indexed to body surface area; LVESV, left ventricular end-systolic volume indexed to body surface area; LVSV, left ventricular stroke volume; LVEF, left ventricular ejection fraction. Statistically significant values are marked with bold*.

* *ANOVA with post hoc Tukey's test if applicable; ^∧^Friedman-ANOVA with post hoc average rank test if applicable;*

a *when normally distributed;*

b *when non-normally distributed*.

**Table 2:** Right ventricular mechanics and 3D motion components in amateur marathon runners.

**Parameter**	**Stage I**	**Stage II**	**Stage III**	**ANOVA#**	***Post-hoc P*** **-value**	
	**Mean** **±** **SD**			***P*-value**	**Stage I vs. II**	**Stage I vs. III**
LEF [%]	22.9 ± 3.2	20.4 ± 3.8	22.2 ± 3.7	>0.05	–	–
REF [%]	23.0 ± 4.5	19.3 ± 4.2	21.2 ± 4.5	**<0.05**	**<0.05**	>0.05
APEF [%]	17.3 ± 3.5	16.4 ± 4.2	17.3 ± 3.5	>0.05	–	–
LEFi	0.46 ± 0.05	0.45 ± 0.08	0.46 ± 0.07	>0.05	–	–
REFi	0.46 ± 0.06	0.42 ± 0.08	0.44 ± 0.07	>0.05	–	–
APEFi	0.35 ± 0.06	0.36 ± 0.07	0.36 ± 0.06	>0.05	–	–

*#Repeated-measures ANOVA with subsequent Tukey's post hoc test for multiple comparisons; Stage I, 2 weeks before the marathon run; Stage II, at the finish line; Stage III, 2 weeks after the competition; LEF, longitudinal ejection fraction; REF, radial ejection fraction; APEF, anteroposterior ejection fraction; LEFi, longitudinal ejection fraction index; REFi, radial ejection fraction index; APEFi, anteroposterior ejection fraction index. Statistically significant values are marked with bold*.

Based on the outcomes of regression modeling, participants reporting higher training distances (expressed as the number of running kilometers per week) showed a greater decline in REF (higher values of the REF change between stages I and II); nevertheless, the relationship was clearly non-linear, following a distinct U-shaped curve with the minimum at around 47 km/week (predicted change of REF between stages I and II with a value of 1.04 ± 5.26). The calculated second-order model explained around 48% of observed REF change and was highly statistically significant (*R*^2^ = 0.4776, *p* = 0.0002). Compared to kilometers/week, the hours/week variable was a worse predictor of REF decline (*R*^2^ = 0.1934, *p* = 0.0290) since, for this parameter, it was not possible to find a model that explained more than 20% of the variation in the change of REF between stages I and II. A greater decline in REF after the competition was also observed in subjects with higher levels of hs-cTnI at stage I (*r* = 0.43, *p* < 0.05).

**Figure 2:**
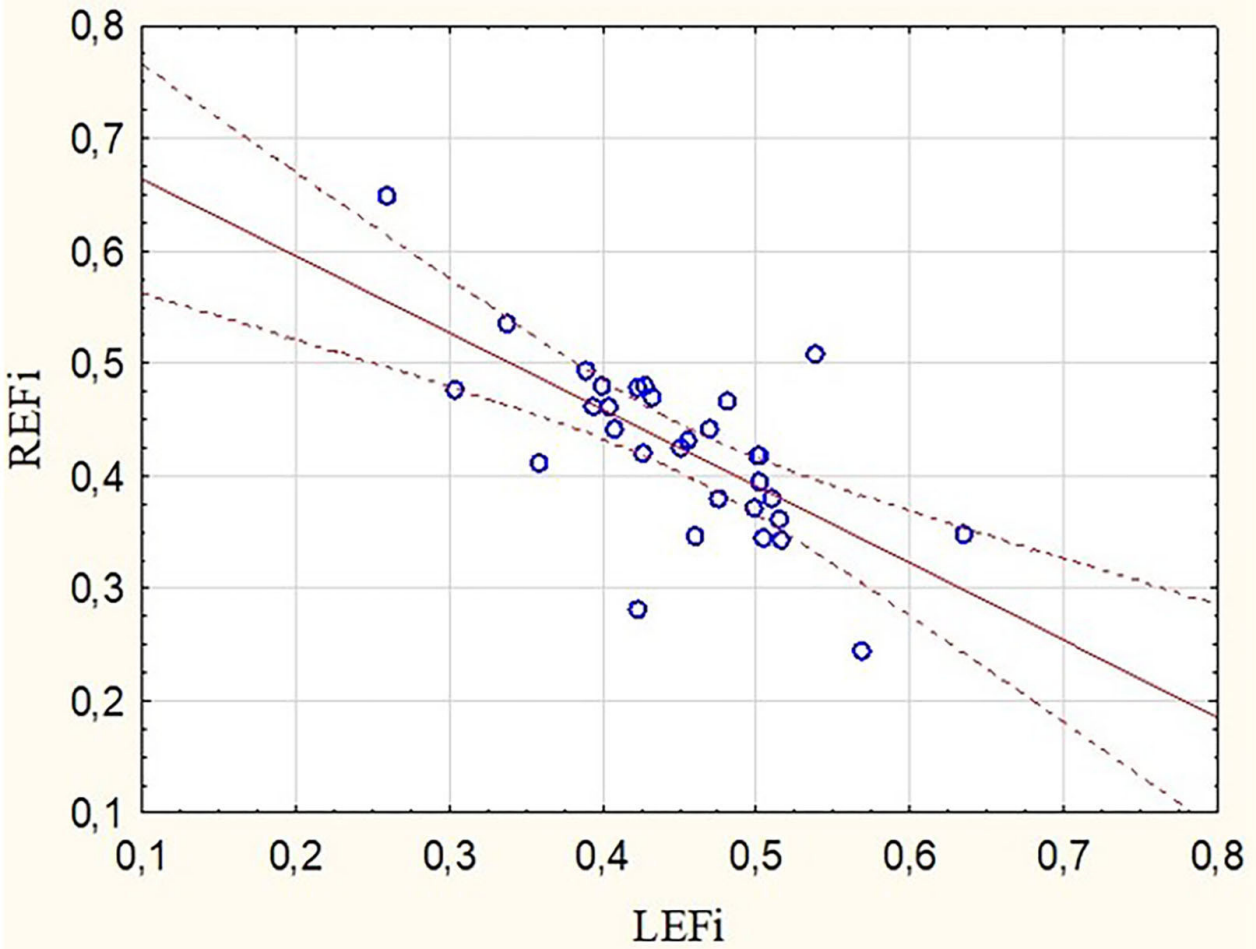
Correlation between radial and longitudinal RVEF indexes obtained after the marathon run (*r* = −0.68, *p* < 0.05). REFi, radial ejection fraction index; LEFi, longitudinal ejection fraction index.

Participants who reported a higher training distance (expressed as running hours per week or as a number of running kilometers during a week) showed higher LEF values (*r* = 0.39 and *r* = 0.38 respectively, *p* < 0.05) at the marathon finishing line. Opposite tendencies were found for the RV radial and longitudinal motion components after the run (Figure 2). There was a negative correlation between REFi and LEFi values (*r* = −0.68, *p* < 0.05) at stage II. Runners with a greater drop in REF at stage II had higher LEFi and LEF after the run as well (*r* = 0.57 and *r* = 0.43, respectively, *p* < 0.05).

**Figure 3:**
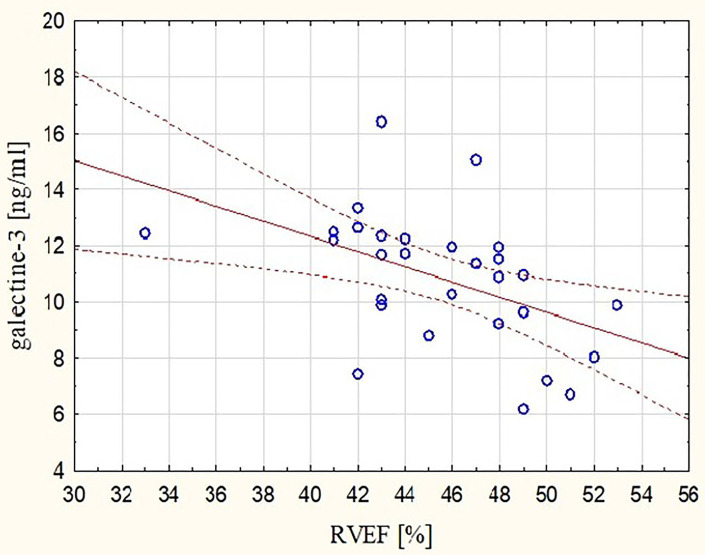
Correlation between post-run RVEF and concentration of galectin-3 (Gal-3) obtained at marathon finishing line (*r* = −0.48, *p* < 0.05). RVEF, right ventricular ejection fraction.

**Figure 4:**
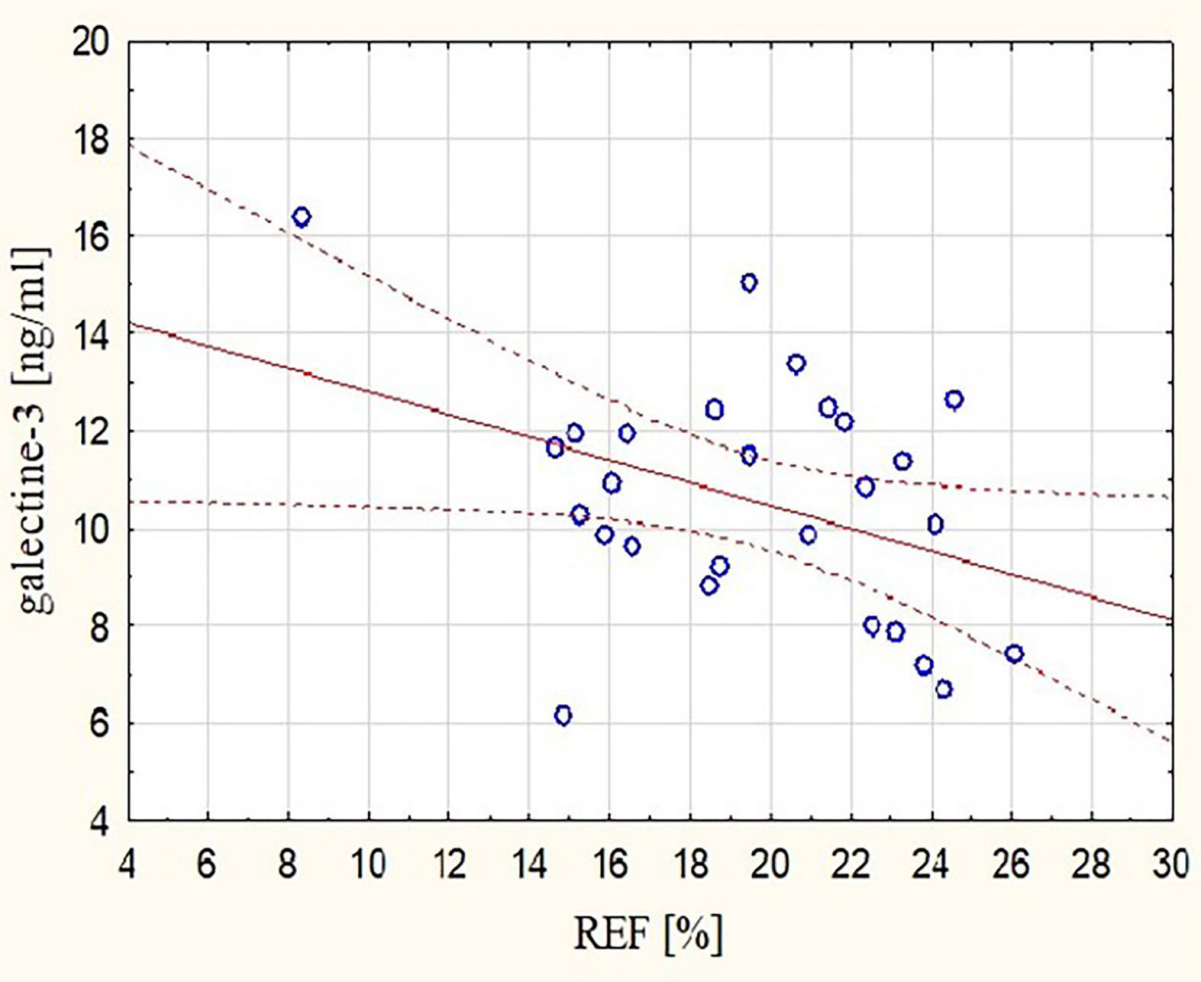
Correlation between post-run RV radial EF and concentration of Gal-3 obtained at marathon finishing line (*r* = −0.39, *p* < 0.05). REF, radial ejection fraction.

**Figure 5:**
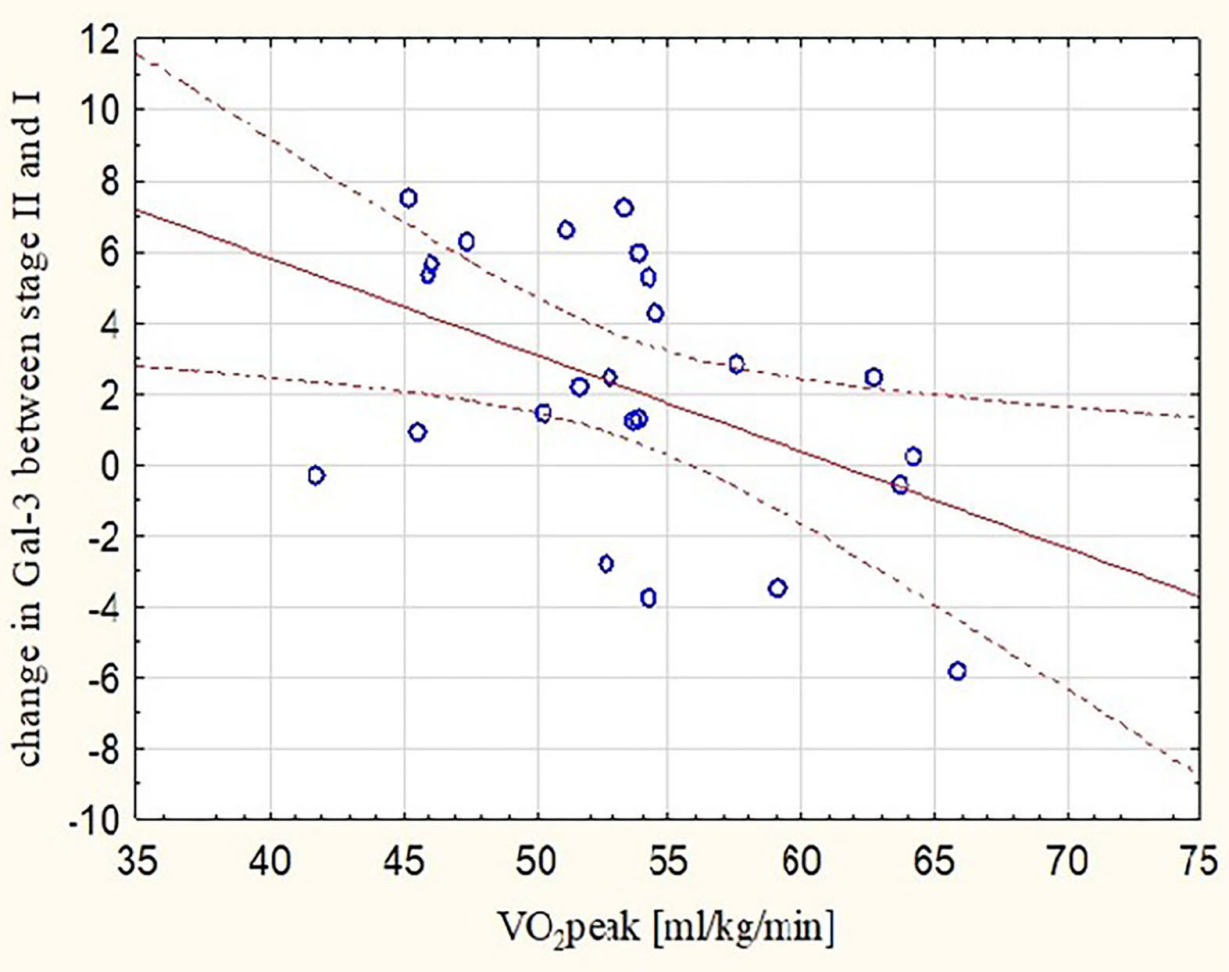
Correlation between peak oxygen uptake and increases in post-marathon Gal-3 concentrations (*r* = −0.47, *p* < 0.05). Gal-3, galectine-3; Stage I-2 weeks before marathon; Stage II-at marathon finish line; VO_2_peak, peak oxygen uptake.

These observations were mirrored by changes in Gal-3 concentrations. After the marathon, higher levels of Gal-3 were found not only in runners with lower RVEF (*r* = −0.48, *p* < 0.05) (Figure 3) but also in those with lower REFi or REF (*r* = −0.44 and *r* = −0.39, respectively, *p* < 0.05) (Figure 4) and higher LEFi at stage II (*r* = 0.43, *p* < 0.05). Greater increases in post-run Gal-3 concentrations were found in less-fit amateurs with lower VO_2_peak values (*r* = −0.47, *p* < 0.05) (Figure 5) and in those who needed more time to finish the competition (*r* = 0.47, *p* < 0.05). After the marathon, positive correlations between LEF or LEFi and hs-cTnI obtained at stage II (*r* = 0.45 and *r* = 0.43, respectively, *p* < 0.05) and between LEFI and H-FABP obtained at stage II (*r* = 0.38, *p* < 0.05) were found. Also, runners with more marked change in LEF had higher raise in hs-cTnI (*r* = 0.62, *p* < 0.05) and H-FABP concentration (*r* = 0.86, *p* < 0.05). The contraction pattern and relative contribution of all RV motion components to global RV systolic function, quantified as LEFi, REFi, and APEFi, did not differ between study stages (Table 2). No significant and strong correlations were found between the other biomarkers collected at stage II and major echocardiographic marathon-induced alterations (the decrease in REF and RVEF).

## Discussion

In this study, we performed a detailed analysis of the impact of running a marathon on RV mechanics in amateur athletes. To the best of our knowledge, this is the first study comparing RV function and its relative motion components before and after running a marathon using the echocardiographic ReVISION method. After the competition, the study participants were found to have a transiently enlarged RV with significantly reduced systolic function. Separate quantification of the RV motion components revealed decreased radial shortening as the main contributor to reduced RV global function with preserved longitudinal and anteroposterior movements. We were able to determine the precise amount of training above which the decrease in radial contraction appeared. The echocardiographic findings of the modified RV mechanics were also reflected on the biochemical level, as significant correlations were found with the profibrotic marker of cardiac remodeling, Gal-3. The post-run deterioration of the radial motion contributor may serve as a novel marker of changed RV systolic function among amateur marathon runners.

While the effects of exercise on the LV have been extensively investigated, the RV has long been called “the dark side of the moon” and has remained unexplored. The variety of 2D ECHO parameters used for RV evaluation in everyday practice refers mainly to longitudinal motion (Lakatos et al., 2017), and measuring RV contractility with these conventional measures seems unrepresentative for the entire RV function. Due to technological advances in recent years, more diagnostic options have become available to evaluate RV functioning; nevertheless, the pathophysiology of the competing RV is still not well understood.

It has previously been shown that various conditions that overburden the RV can induce significant changes in its contractile pattern and that one motion contributor may become more important to the EF than others. Similarly, in patients with tricuspid or pulmonary valve regurgitation, an increased longitudinal RV motion following volume overload was reported (Kovács et al., 2019). Therefore, there may be a greater decrease in one motion component over the others in some situations, reflecting a marker of RV dysfunction, as in pulmonary hypertension or pulmonary embolism where a pressure overload causes a greater reduction in radial vs. longitudinal shortening, which becomes supernormal (Lakatos et al., 2017, 2020).

There are some similarities between patients with elevated pulmonary arterial systolic pressure (PASP) and subsequent RV overload and marathon runners in terms of the reduced radial shortening observed in both groups.

While running a marathon, the cardiac output (CO) increases even up to 40 L/min, which is seven times higher than in normal resting conditions (La Gerche et al., 2014). Bearing in mind the simplified Poiseuille's equation that illuminates the functioning of the circulatory system, such an increase in CO must result in increased vascular pressure unless it is lowered by a drop in resistance (La Gerche et al., 2014). The RV normally pumps blood against a low-resistance and low-pressure pulmonary circuit; however, its load changes dramatically during intense exercise, as the drop in pulmonary vascular resistance seems to be relatively smaller than in the systemic circulation (La Gerche et al., 2014). Consequently, as PASP increases significantly, pressure overload is added, proportionally with augmented CO, and a subsequent increase in RV wall stress is observed (La Gerche et al., 2011, 2014). Although the raise in PASP is transient and normalizes briefly after the exercise (Wierzbowska-Drabik et al., 2019), it causes increased RV work, and as shown in this study, the acute effects following a marathon run manifest as an increase in RV volumes, a decrease in radial contraction, and a deterioration in global RV systolic function. Overall, the impact of running a marathon is mainly on the RV, whereas the LV seems to cope well.

Among the amateur athletes, the post-run impairment of RV function was transient and completely normalized in 2 weeks of observation. Nevertheless, in cases of sustained, repetitive exercising, functional RV changes can consolidate into RV remodeling that is not entirely physiological. In a rat model, after 4 months of forced training, the exercise-induced changes in ventricular function resulted in increased fibrosis in the RV and both atria followed by increased susceptibility to ventricular arrhythmias (Benito et al., 2011).

The marathon effect was also manifested by noticeably elevated concentrations of hs-cTnI, BNP, and stress-related biomarkers, such as GDF-15 and Gal-3 (Kaleta-Duss et al., 2020). Often considered a determinant of myocardial injury, such a “biochemical and biomarker storm” may prompt the creation of fibrotic deposits, mainly in the most strained cardiac chambers. Although the direct proarrhythmic significance in humans has not been proved yet, the incidence of fibrotic changes was higher in marathon runners than in age-matched controls (12 vs. 4%) (Breuckmann et al., 2009). It is noted that the prevalence of myocardial fibrosis, which was found in 50% of veteran endurance runners, was related to the years of training and the number of completed endurance competitions (Wilson et al., 2011). Athletes with delayed gadolinium enhancement in the interventricular septum near the RV attachment had greater RV volume, lower RVEF, and a longer history of endurance sports participation (La Gerche et al., 2012). Interestingly, ventricular arrhythmias recorded in athletes usually originated from the RV (Heidbüchel et al., 2003). Although the data on the eventual proarrhythmic effect of endurance exercise are limited, the high prevalence of atrial fibrillation is a well-known phenomenon among athletes in whom the incidence of arrhythmia is associated with left atrial remodeling and, typically, increased atrial volume (Elliott et al., 2018). Unquestionably, sports activity evokes arrhythmias in individuals with preexisting cardiac disease, such as RV arrhythmogenic cardiomyopathy or hypertrophic cardiomyopathy (Pelliccia et al., 2021).

Nevertheless, the highest risk of acute events, such as cardiac arrest, exercise-related collapse, and chest pain, is linked with middle-aged male athletes who finish marathon runs within 3–4 h (Sharma, 2017). Although frequently exhibiting a cardiovascular risk typical of their peers, the middle-aged group of amateur athletes typically aims to achieve their best results during sports competitions. The question is whether they are adequately prepared to run a marathon in terms of cumulative RV fatigue and the risk of being overtrained.

**Figure 6:**
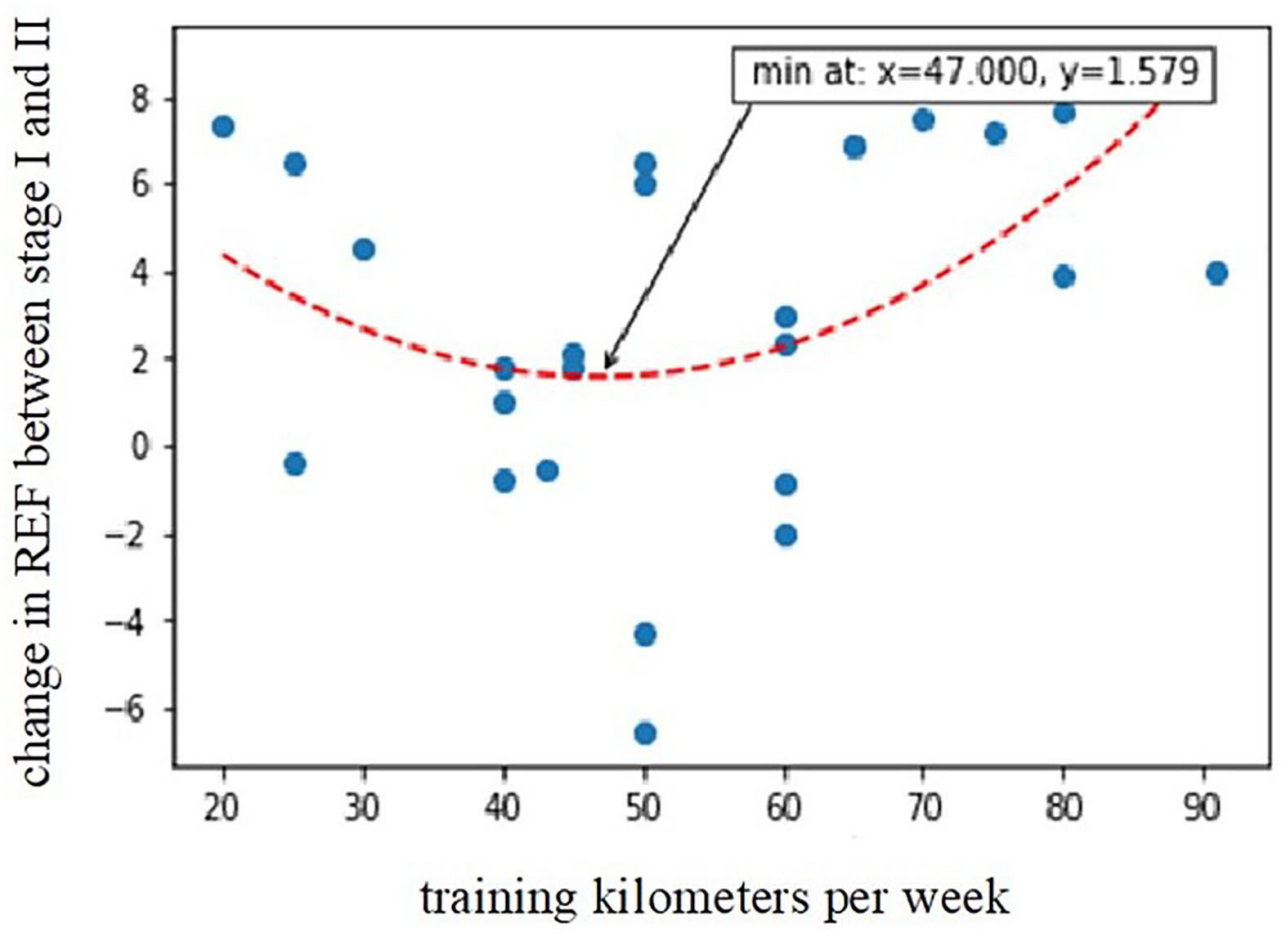
Relationship between training kilometers per week during the preparation period and post-marathon decline in radial EF (*R*^2^ = 0.4776, *p* = 0.0002). REF, radial ejection fraction; Stage I-2 weeks before marathon; Stage II-at marthon finish line.

In this study, higher baseline hs-cTnI levels were associated with a greater decline in post-marathon RV radial shortening. Moreover, we proved that the amount of training in the preparation period is important in terms of observed postrace REF changes. Exercising of more than 47 km/week was connected with a prominent postrace reduction in RV radial contraction. However, not only too much but also very minimal training is meaningful and best described by the “U-shaped curve” (Figure 6). Marathon runners who train less are at high risk of marathon-induced myocardial injury because they develop higher pulmonary pressure, greater RV dysfunction, and elevated cardiac troponin T and N-terminal pro-brain natriuretic peptide (Neilan et al., 2006). In our study, those who were less fit (who had a lower VO_2_peak in CPET) had higher post-competition plasma concentrations of Gal-3.

An increased concentration of Gal-3 involved in the processes of fibrosis and inflammation is found in acute and chronic heart failure (Coburn and Frishman, 2014). In patients with reduced LVEF, it correlates with the geometry and function of RV and PASP (Zaborska et al., 2020). Gal-3 is considered to be an important factor contributing to cardiopulmonary remodeling in pulmonary hypertension (Barman et al., 2019). In the general population, Gal-3 concentrations predict all-cause mortality (de Boer et al., 2012). In the studied amateur runners, the post-marathon Gal-3 measurements accurately reflected the RV performance, and higher Gal-3 concentrations were found in marathoners with lower global RV function and decreased radial shortening at stage II. These results may suggest a possible link between exercise-induced RV fatigue and myocardial fibrosis, although more research is needed to prove such a relationship.

As many studies have reported preserved RV function in athletes (Leischik et al., 2020), it is likely that a sufficiently long low-intensity preparation period determines the RV response to endurance exercise. The time required for the RV to adapt to the increased overload is not known and possibly depends on the individual. The increased RV dimensions with preserved RVEF are known cardiac adaptive changes called the “athlete's heart” features (Sanz-de la Garza et al., 2020).

In this study, we have shown that among amateur athletes, exhausting exercise acutely but reversibly alters RV radial shortening. It is possible that the long-term RV adaptation also involves persistent changes in the RV contractile pattern and the constant redistribution of motion components. Undoubtedly, the decrease in one systolic contributor can be compensated for by the increased shortening of others, and the global RV function may be preserved. Similarly, heart-transplant recipients, in whom radial shortening is usually superior to longitudinal and reduced 2D parameters, do not indicate RV failure (Lakatos et al., 2018b). Interestingly, after the marathon, we observed different trends in radial and longitudinal motion and negative correlations between them, which was also reflected in Gal-3 concentrations. In another study performed only in resting conditions, top-level water polo players were compared with sedentary controls, and a significant shift in RV contraction pattern was reported with increased longitudinal vs. radial motion (Lakatos et al., 2018a). The more trained the players, the more evident the RV motion changes they presented (Lakatos et al., 2018a). Therefore, the question arises whether such a permanent change in the mechanics of RV contraction may also develop in the group of recreational runners; however, this requires follow-up observation. Further studies are also necessary to determine whether the reduction in the radial motion contributor is a new feature of the “athlete's heart” or a marker of the onset of RV dysfunction.

## Conclusion

Completing marathon results in significant RV changes in amateur athletes, with decreased RV contractility and increased RV volumes, and less if any effect on the LV function. The drop in global RV function occurs due to a transient decline in its radial shortening, with preserved longitudinal and anteroposterior motion components. The ReVISION method enables a comprehensive analysis of the mechanics of RV contraction and allows insight into the physiology of the competing heart. The observed post-competition macroscopic remodeling of the RV is also reflected at the biochemical level, as the echocardiographic findings correlate with the changes in concentrations of a so-called marker of fibrosis, Gal-3, and a more profound RV dysfunction appears in runners with higher Gal-3 levels. The importance of adequate training preparation for marathon participation seems unquestionable. The thesis of cumulative RV damage is supported here, as a decrease in radial contraction occurred in those who have been probably under- and overtrained, and higher Gal-3 levels after the run were found in those who were less fit.

## Data Availability Statement

The raw data supporting the conclusions of this article will be made available by the authors, without undue reservation.

## Ethics Statement

The studies involving human participants were reviewed and approved by Independent Bioethics Commission for Research of the Medical University of Gdansk. The patients/participants provided their written informed consent to participate in this study.

## Author Contributions

ZL-P, AK-D, EL, and AD-K: contributed to the conceptualization and design of the study. ZL-P, AK-D, EL, MK, AF, PS, RG, GR, and AD-K: managed investigation, analysis, and methodology. ZL-P, AK-D, and AD-K: organized the database. ZL-P and AD-K: performed the statistical analysis. ZL-P: wrote the first draft of the manuscript. EL and AD-K: supervised writing the manuscript. All authors contributed to manuscript revision, read, and approved the submitted version.

## Funding

The publication of this study was supported by the project POWR.03.05.00-00-z082/18 and co-financed by the European Union through the European Social Fund under the Operational Programme Knowledge Education Development 2014–2020.

## Conflict of Interest

The authors declare that the research was conducted in the absence of any commercial or financial relationships that could be construed as a potential conflict of interest.

## Publisher's Note

All claims expressed in this article are solely those of the authors and do not necessarily represent those of their affiliated organizations, or those of the publisher, the editors and the reviewers. Any product that may be evaluated in this article, or claim that may be made by its manufacturer, is not guaranteed or endorsed by the publisher.
